# Alzheimer’s disease: natural products as inhibitors of neuroinflammation

**DOI:** 10.1007/s10787-020-00751-1

**Published:** 2020-09-15

**Authors:** Olumayokun A. Olajide, Satyajit D. Sarker

**Affiliations:** 1 Department of Pharmacy, School of Applied Sciences, University of Huddersfield, Queensgate, Huddersfield, HD1 3DH UK; 2 Centre for Natural Products Discovery, School of Pharmacy and Biomolecular Sciences, Liverpool John Moores University, James Parsons Building, Byrom Street, Liverpool, L3 3AF UK

**Keywords:** Alkaloids, Marine natural products, Polyphenols, NF-κB, Terpenes

## Abstract

Alzheimer’s disease (AD) is the most common form of dementia and affects 44 million people worldwide. New emerging evidence from pre-clinical and clinical investigations shows that neuroinflammation is a major pathological component of AD suggesting that anti-inflammatory strategies are important in delaying the onset or slowing the progression of the disease. However, efforts to employ current anti-inflammatory agents in AD clinical trials have produced limited success. Consequently, there is a need to explore anti-inflammatory natural products, which target neuroinflammatory pathways relevant to AD pathogenesis. This review summarises important druggable molecular targets of neuroinflammation and presents classes of anti-neuroinflammatory natural products with potentials for preventing and reducing symptoms of AD.

## Introduction

Alzheimer’s disease (AD) is the most common form of dementia. AD has been reported to affect about 44 million people globally, and is estimated to triple by 2050 due to general population ageing (Prince et al. 2014). The main pathological features of AD include extracellular amyloid-beta (Aβ) plaques and intracellular neurofibrillary tangles (Bloom 2014). Studies have established that there is a strong correlation between symptoms of AD and the accumulation of these plaques and tangles due to their ability to induce neurodegeneration that mediates loss of memory and cognition.

Interestingly, new emerging evidence continues to demonstrate that neuroinflammation is also a major pathological component of AD (Webers et al. 2020). Several reports of animal experiments and clinical studies have provided strong links between neuroinflammation and AD pathogenesis. Increasing evidence from several studies revealed that inflammatory responses in the brain are a major contributor to the pathogenesis of AD (Heppner et al. 2015; Fu et al. 2019). In fact, high levels of pro-inflammatory mediators have been detected in the brain of AD patients (Hesse et al. 2016). Furthermore, neuroinflammation which is characterised by activation of brain-resident macrophages with the resultant hyper-secretion of pro-inflammatory cytokines and chemokines such as interleukin-1beta (IL-1β), interleukin-6 (IL-6), tumour necrosis factor-alpha (TNFα), interleukin-8 (IL-8), transforming growth factor-β (TGF-β) and macrophage inflammatory protein-1α (MIP-1α) is a well-documented consequence of high levels of insoluble forms of Aβ (Akiyama et al. 2000). Consequently, anti-inflammatory strategies have the potential to delay the onset or slow the progression of the disease.

It is now well-established that the transcription factor nuclear factor-kappa B (NF-κB) plays a major role in neuroinflammation-mediated AD. NF-κB is a master regulator of inflammatory gene transcription and has been shown to be expressed in the brains of AD patients (Boissière et al. 1997; Liao et al. 2016). NF-κB has also been proposed as a molecular mechanism underlying the development of some sporadic cases of AD (Chen et al. 2012). These reports linking NF-κB to AD strengthen the role of neuroinflammation in AD.

A number of molecular mechanisms and cellular signalling pathways have been proposed to contribute to neuroinflammation in AD. Some of these mechanisms are known to be under the direct influence of NF-κB, while others have been reported to cross-talk with this transcription factor in a manner that makes them a molecular target for drug action in AD therapeutics.

The signalling pathways involving the mitogen activated protein kinases (MAPKs) have been strongly linked to neuroinflammation and AD. Of the MAPKs, the p38 MAPK has been implicated in neuroinflammation. Evidence linking p38 MAPK to neuroinflammation was put forward by Kim and Choi (2015), who suggested that exposure of microglia to Aβ induces microglial activation with the subsequent production of neurotoxic pro-inflammatory cytokines and reactive oxygen species, which in turn activate p38 MAPK signalling. Further reports show that Aβ-induced oxidative stress results in the activation of p38 MAPK with the resultant tau hyperphosphorylation (Giraldo et al. 2014). Recent reports have also suggested that p38 MAPK plays a role in neuroinflammation and AD due to its ability to activate NF-κB (Kheiri et al. 2018), thus making it a potential molecular target for novel AD treatment.

The nuclear factor E2-related factor 2 (Nrf2) is a transcription factor that regulates phase II antioxidant response mechanisms in response to oxidative stress. Emerging evidence links activation of the Nrf2 protective mechanism to anti-inflammatory effects involving NF-κB (Nair et al. 2008; Sandberg et al. 2014). Specifically, NF-κB is a known negative regulator of Nrf2 (Liu et al. 2008; Kim et al. 2010a, b; Yu et al. 2011). Rojo et al. (2010) demonstrated that an activation of the microglia in Nrf2-deficient animals is accompanied by increased levels of pro-inflammatory cyclooxygenase-2 (COX-2), inducible nitric oxide synthases (iNOS), IL-6, and TNFα. To confirm these observations, Ramsey et al. (2007) reported that brains from AD patients have decreased levels of Nrf2 in the hippocampus. Consequently, there is an increasing interest in pharmacological activators of Nrf2 to activate or restore its protective mechanisms (Sandberg et al. 2014).

Research findings implicating neuroinflammation in AD have resulted in pre-clinical and clinical investigations of NSAIDs and other anti-inflammatory agents as potential therapeutic strategies for AD (McGeer et al. 2016; Cuello, 2017). However, efforts to use current anti-inflammatory agents in AD clinical trials have not been successful. Investigations showed that anti-inflammatory drugs failed to delay or reduce the pathological symptoms of patients with mild cognitive impairment or AD (Fu et al. 2019). Most of the anti-inflammatory drugs which have been investigated in clinical trials for AD are known to target specific single inflammatory mechanisms. It was therefore not surprising that these drugs failed in clinical trials, given the multi-faceted nature of AD pathology. The multi-target approach is an alternative strategy in evaluating anti-inflammatory drugs for effectiveness in slowing the progression of AD.

Some natural products are able to produce multi-target anti-inflammatory activity in AD through modulation of multiple signalling pathways. It has been proposed that the multi-pharmacological actions of black and green tea polyphenols could be valuable in the treatment of neurodegenerative disorders including AD (Mandel et al. 2011, 2012). Inhibition of neuroinflammation by natural products has also been linked to their ability to produce anti-amyloid effect. Apigenin is an anti-inflammatory natural product which showed effects on APP processing and preventing Aβ burden through down-regulation of BACE1 levels, the relief of Aβ deposition, and the decrease of insoluble Aβ levels (Zhao et al. 2013). It is worth evaluating other anti-inflammatory natural products with multi-target anti-inflammatory activity as potential candidates for AD therapeutics.

## Natural product inhibitors of neuroinflammation

Several natural products have been reported to produce anti-neuroinflammatory activity through mechanisms involving inhibition of microglia activation, reduction of the release of pro-inflammatory cytokines from activated microglia, or through inhibition of NF-κB and p38 MAPK activation. Other natural products produce marked activation of Nrf2, a mechanism which has been shown to contribute at least in part to their anti-neuroinflammatory activity. This review will highlight some of our investigations and those reported by other investigators on the main classes of natural products with promising therapeutic potentials for inhibiting neuroinflammation.

### Alkaloids

**Fig. 1:**
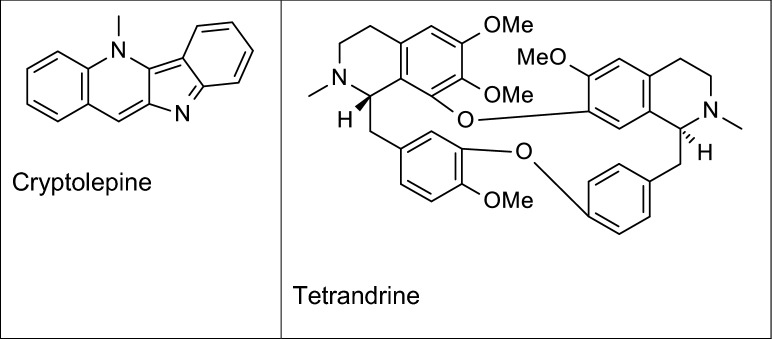
Examples of some anti-neuroinflammatory alkaloids

Alkaloids are pharmacologically-active secondary metabolites consisting of nitrogen, often as an integral part of the ring (Ziegler and Facchini 2008; Nahar and Sarker 2019) and exist as either proto-alkaloids (nitrogen-containing but not heterocyclic in structure) or true alkaloids (nitrogen-containing heterocyclic compounds) (Rosa et al. 2007). Alkaloids (Fig. 1) have been linked to a wide variety of pharmacological activities, including anti-inflammatory effects.

With regards to anti-inflammatory activity in the CNS, our investigations revealed that the alkaloid cryptolepine found in *Cryptolepis sanguinolenta* produced a reduction in the levels of TNFα, IL-6, IL-1β, NO, and PGE_2_ in LPS-stimulated rat microglia. There are currently no studies to determine demonstrating anti-inflammatory effects of cryptolepine on microglia stimulated with either amyloid beta. We also observed reductions in protein and mRNA levels of COX-2 and iNOS and further demonstrated that the effects of the compound are mediated through blocking activation of NF-κB, p38 MAPK in the microglia (Olajide et al. 2013). The effects of cryptolepine in animal models of neuroinflammation or AD are yet to determined. However, we have shown that this alkaloid produced anti-inflammatory activity in animal models of peripheral inflammation (Olajide et al. 2009). A major limitation in the development of this alkaloid in the treatment of AD is related to its cytotoxicity due to its ability to cause DNA damage (Gopalan et al. 2011).

In similar fashion, tetrandrine (a bisbenzylisoquinoline alkaloid isolated from Radix *Stephania tetrandra*) has shown promising NF-κB-mediated anti-inflammatory activity in BV-2 microglia activated with fibrillar amyloid beta and reduced hippocampal neuroinflammation by inhibiting NF-κB activation in a rat model of AD induced by amyloid beta (He et al. 2011a, b). While there are no studies demonstrating the clinical efficacy of tetrandrine in AD, it has been suggested that this alkaloid is able to permeate the blood–brain barrier to provide benefits in stroke, due to its lipophilic nature (Chen et al. 2011). A potential limitation to developing tetrandrine for clinical use is related to its ability to produce significant unwanted effects in the cardiovascular system. Tetrandrine is a known calcium channel blocker (King et al. 1988) and has a potential to induce reduction in peripheral resistance as well as decreasing heart rate and cardiac contractility, all of which could result in a reduction in blood pressure and induction of arrhythmias.

### Flavonoids and other polyphenols

**Fig. 2:**
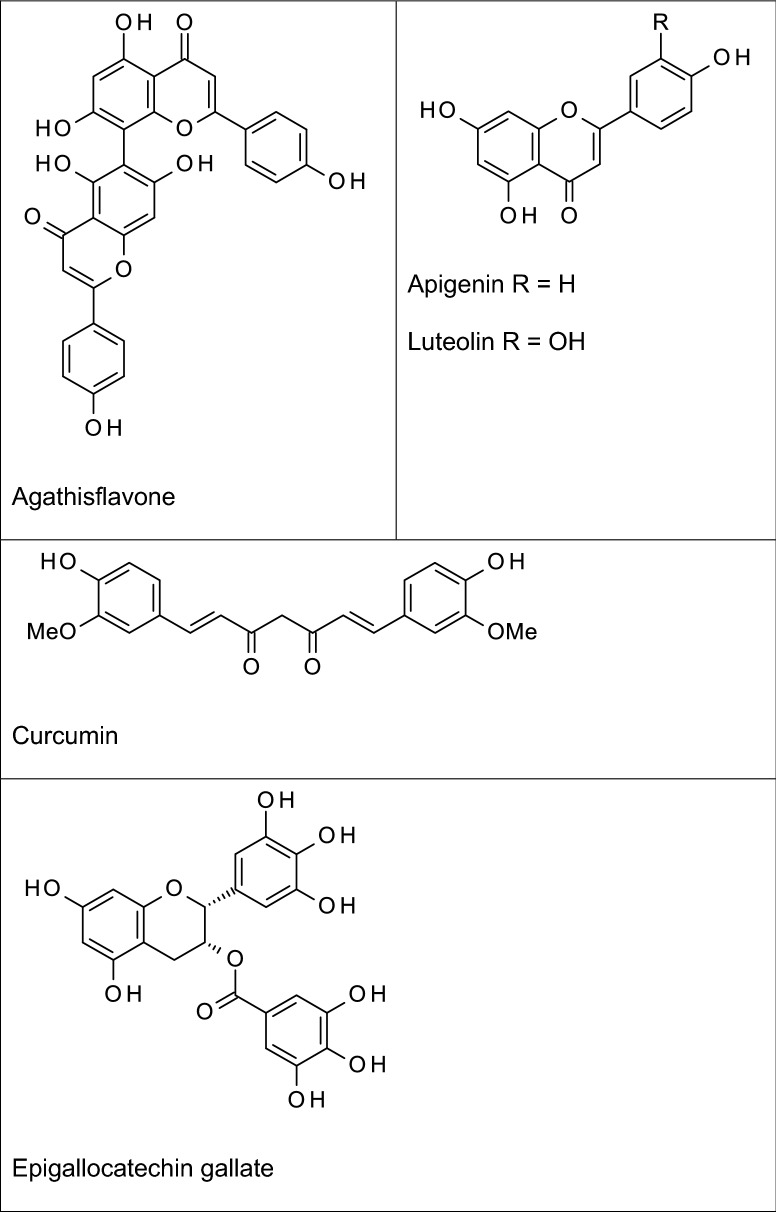

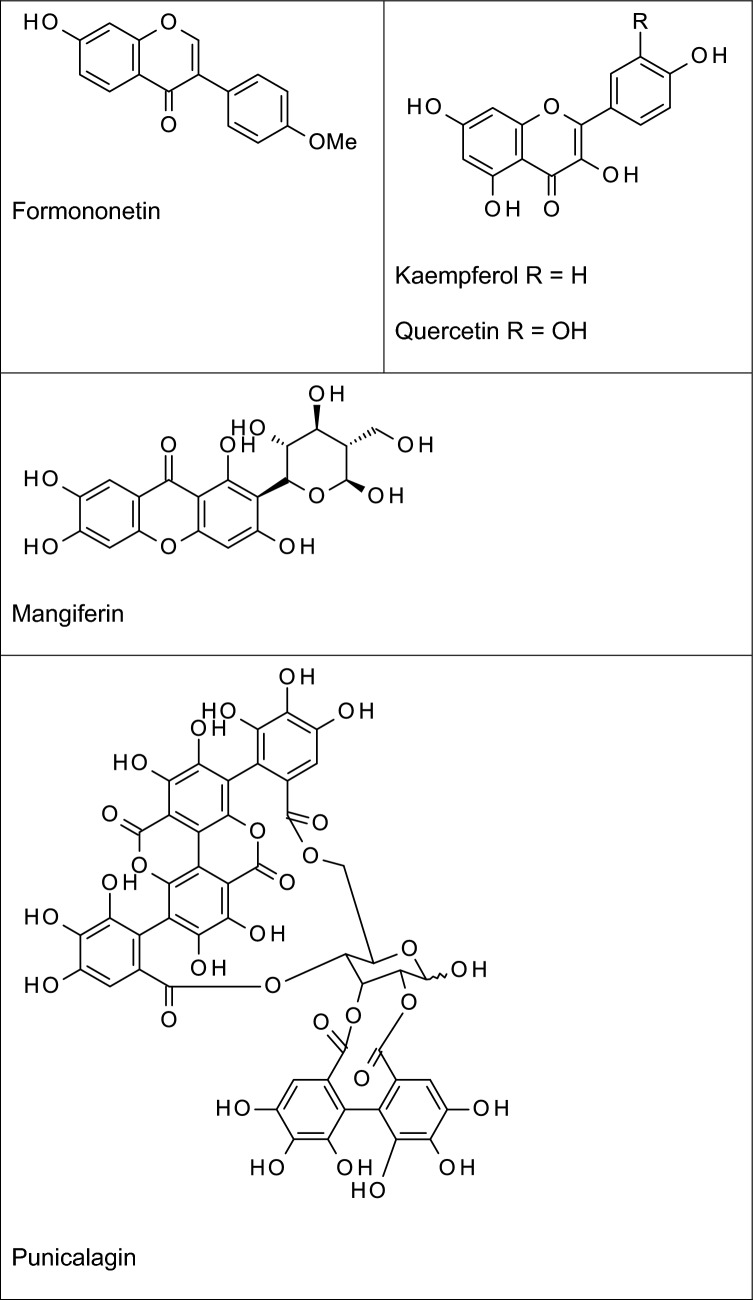

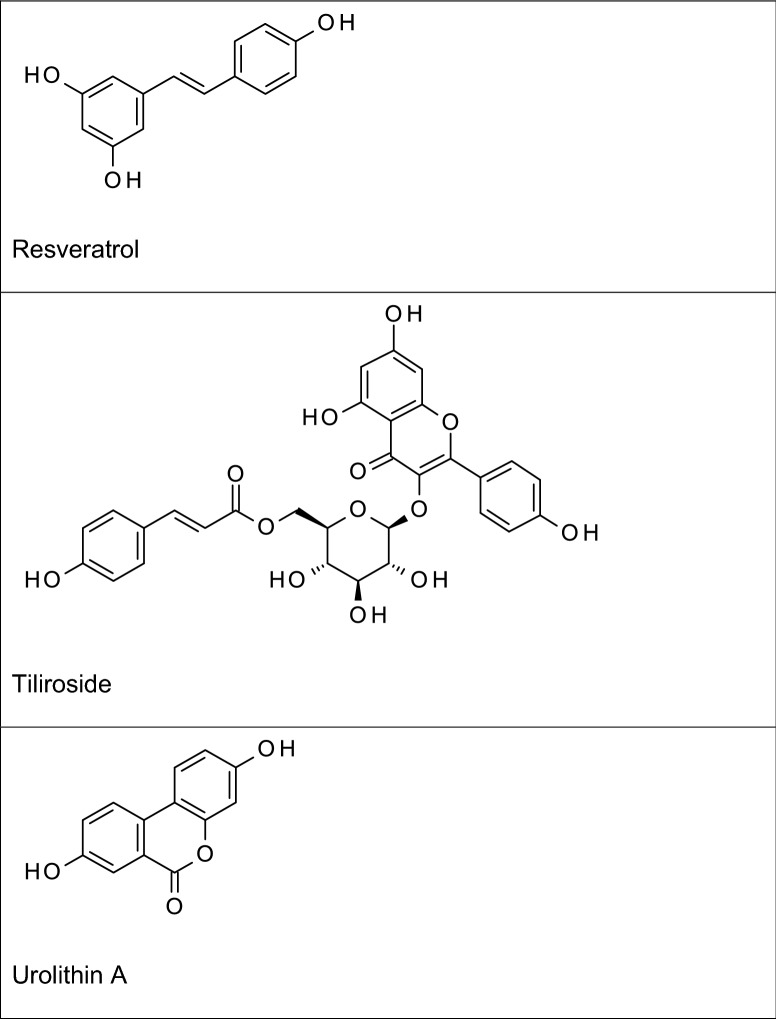
Examples of some anti-neuroinflammatory flavonoids and polyphenols

Flavonoids are naturally occurring phenolic compounds that can be structurally classified as anthocyanins, catechins, flavones, flavonols, flavanols, flavanones, and isoflavonoids. They are found in abundance in flowers, fruits, barks, roots, stems, tea, wine and vegetables (Nahar and Sarker 2019). Several flavonoids and polyphenolic compounds have been shown to possess anti-neuroinflammatory activity (Fig. 2).

Kaempferol is a flavonol which has been shown to inhibit neuroinflammation by reducing LPS-induced production of pro-inflammatory mediators in BV2 microglial cells through mechanisms involving NF-κB and p38 MAPK (Park et al. 2011). A related glycosidic flavonoid to kaempferol, tiliroside (contained in plants such as rosehip, linden and strawberry) was demonstrated in our studies to inhibit neuroinflammation in BV-2 microglia through multiple mechanisms including attenuation of NF-κB and p38 MAPK, in addition to activating Nrf2 (Velagapudi et al. 2014, 2018a, b). Neither kaempferol nor tiliroside has been investigated for clinical efficacy in humans. Apigenin (4′,5,7-trihydroxyflavone), structurally similar to kaempferol with just one less –OH group at C-3 is a flavone found in chamomile, celery and parsley, and many other plants. Studies reported by Rezai-Zadeh et al. (2008) showed that treatment of interferon gamma-activated microglia with apigenin resulted in a decrease in the production of pro-inflammatory IL-6 and TNFα through mechanisms involving STAT1. Further evidence of the anti-inflammatory activity of this flavonoid was provided in investigations showing reductions in iNOS/NO and PGE_2_/COX-2 in activated microglia (Choi et al. 2014). Animal studies have also suggested that the anti-inflammatory activity of apigenin may contribute to its neuroprotective activity in models of AD. Treatment of mice with apigenin improved spatial learning and memory following amnesia induction with Aβ_25–35_ (Liu et al. 2011). It is widely known that one of the mechanisms involved in Aβ-induced neurodegeneration involves neuroinflammation (Ralay Ranaivo et al. 2006), suggesting that the neuroprotective activity reported by Liu et al. (2011) may be related to anti-inflammatory action of the compound.

Quercetin, a similar flavonol to kaempferol with an extra hydroxyl group at C-3′ (B ring) is a ubiquitous anti-inflammatory and antioxidant natural product that is found in many fruits, vegetables, and seeds. Treatment with quercetin reduced iNOS-mediated NO production in LPS-stimulated BV-2 microglia through mechanisms involving suppression of NF-κB activation (Kang et al. 2013). These authors further demonstrated that the antioxidant transcription factor, Nrf2 is required for the anti-inflammatory effect of quercetin (Kang et al. 2013). These observations were supported by studies linking inhibition of neuroinflammation by quercetin to potential cross-talk between MAPKs and Nrf2 (Sun et al. 2015).

The anti-inflammatory activity of quercetin has been linked to its effect on cognitive function in APP/PS1 mouse model of AD. Investigations by Lv et al. (2018) demonstrated that quercetin treatment of APP/PS1 mice significantly reduced Aβ plaques, p-Tau and neuroinflammation. The antioxidant activity of quercetin, through activation of Nrf2 and the subsequent anti-inflammatory effect in the microglia are significant in neuroprotection and therapeutic benefits in AD. There are no studies showing clinical efficacy of quercetin in AD. This may be due to its poor permeation of the BBB. Similar anti-inflammatory/neuroprotective profiles have been reported for the related flavone luteolin (Burton et al. 2016; Yao et al. 2018).

Epigallocatechin-3-gallate (EGCG) is a flavanol found mainly in green tea (*Camellia sinensis*). Studies have suggested that the anti-amyloidogenic and neuroprotective actions of this flavonoid may be due to its ability to inhibit neuroinflammation. Experiments conducted in vitro showed that EGCG could reduce TNFα, IL-1β, IL-6, iNOS levels in Aβ-stimulated EOC13.31 microglia through mechanisms involving NF-κB (Wei et al. 2016). In animals, Lee et al. (2013) reported that EGCG prevented memory impairment and reduced the levels Aβ generation and neurotoxicity in mice following systemic injection of lipopolysaccharide (LPS). Similar observations were made in studies reported by Seong et al. (2016) who further linked the anti-neuroinflammatory activity of this compound to inhibition of NF-κB activation. In vivo experiments have also shown that EGCG produced neuroprotection in animal models of AD. For example, intraperitoneal injection of 12-month-old Tg2576 mice with 20 mg/kg EGCG resulted in decreased levels of Aβ as well as plaque load in the brain. Similar observations were made following oral administration of the compound to TgCRND8 (Tg) mice (Walker et al. 2015).

Human trials to evaluate the efficacy of EGCG in improving cognitive function have not reflected results achieved in vitro and in animal models of AD. For example, administration of 300 mg EGCG to healthy volunteers was shown to increase cerebral activity (as evidenced by an increase in alpha, beta and theta activities in the brain) without a corresponding effect on task performance (Scholey et al. 2012). Furthermore, a double‐blind, placebo‐controlled, crossover investigation of a single oral dose of 135 mg EGCG on cognitive performance, mood and cerebral blood flow (CBF) in healthy human adults did not show any significant effects on mood and cognition, in comparison with placebo (Wightman et al. 2012). The translational gap between bioactivity of EGCG in vitro and in animal studies and its effects in human trials is possibly associated with low oral bioavailability of the compound on the one hand, as well as different metabolism between animals and humans on the other hand (Mähler et al. 2013). More long-term human trials are necessary to establish the effects of EGCG on cognitive performance. Investigations to compare oral bioavailability and BBB penetration of EGCG in mice and human subjects will throw more light on the apparent lack of efficacy in human studies. EGCG consumption has been associated with hepatoxicity (Navarro et al. 2013; Hu et al. 2018). This effect of the compound needs to be taken into consideration in future clinical development for AD therapeutics.

Investigations on the pomegranate fruit polyphenol punicalagin revealed that the compound produced significant inhibition of neuroinflammation in LPS-activated rat primary microglia through interference with mechanisms resulting in activation of NF-κB (Olajide et al. 2014). Interestingly, one of its gut-derived metabolites urolithin A, showed similar effects on LPS-activated BV-2 microglia (Velagapudi et al. 2019). Urolithin A has also produced neuroprotection by blocking memory impairment and neuroinflammation in APP/PS1 mice (Gong et al. 2019) and in a *Caenorhabditis elegans* model (Yuan et al. 2016; Fang et al. 2019). Based on the published literature no studies in humans have been conducted on pomegranate polyphenols or their gut-derived metabolites with respect to cognitive performance or other therapeutic measures of AD.

Mangiferin is a naturally occurring glucosylxanthone found in the stem bark and leaves of mango plant (*Mangifera indica*). Investigations in rat primary microglia revealed that mangiferin could inhibit COX-2 expression and prostaglandin E2 (PGE_2_) production following activation with LPS (Bhatia et al. 2008). In vivo experiments showed that mangiferin diminished neuroinflammation and improved cognitive deficits in APP/PS1 mice (Infante-Garcia et al. 2017). Based on published literature no human studies have demonstrated the efficacy of mangiferin in therapeutic endpoints of AD.

Resveratrol is a polyphenol found in grapes and berries. This stilbene is reputed with various pharmacological activities and has been widely studied as potential treatment for diverse disorders. Several pharmacological studies have demonstrated anti-neuroinflammatory/neuroprotective effects of resveratrol in in vitro and in animal models. The first indication of inhibition of neuroinflammation by resveratrol was reported by Candelario-Jalil et al. (2007) who provided evidence that this compound inhibited PGE_2_ production and free radical formation in LPS-activated primary rat microglia. This study further revealed that resveratrol was the first known inhibitor which specifically prevents microsomal prostaglandin E synthase-1 (mPGES-1) expression without affecting COX-2. Subsequent studies by Abraham and Johnson (2009) demonstrated that resveratrol consumption resulted in reduction of LPS-induced IL-1β in plasma and IL-1β mRNA in the hippocampus of aged mice, as well as in cultured BV-2 microglia. Other studies over the last few years have increased the evidence demonstrating inhibition of neuroinflammation and neuroinflammation-mediated neuronal damage by resveratrol (Lu et al. 2010; Zhang et al. 2013; Potter et al. 2013; Wang et al. 2015; Yao et al. 2015). It is noteworthy that a recent study by Sun et al. (2019) provided a new evidence linking inhibition of neuroinflammation by resveratrol to its ability to rescue tau-induced cognitive deficits and neuropathology in a mouse model of AD. In that study, treatment with resveratrol rescued cognitive deficits, reduced levels of phosphorylated tau, prevented neuroinflammation and synapse loss in the brains of mice. These pre-clinical reports on resveratrol are promising and warrant further clinical evaluation.

In a clinical trial, treatment of mild to moderate AD patients with resveratrol resulted in the decline of cerebrospinal fluid amyloid beta, which is an AD biomarker. This was accompanied by reduction in biomarkers of neuroinflammation (Moussa et al. 2017). However, a pilot study to study the effects of chronic resveratrol use on cognitive function in elderly subjects revealed selectively improved psychomotor speed without significantly affecting other domains of cognitive function (Anton et al. 2018). These findings providing modest clinical evidence for the efficacy of resveratrol in AD is possibly connected to low oral bioavailability of the compound. Larger placebo-controlled, randomised trials with bioavailable formulations of resveratrol are required to throw more light on the efficacy of the compound in humans.

Curcumin found in *Curcuma longa* (turmeric) is perhaps the most investigated natural neuroprotective polyphenol for treating AD. It has been linked to a diverse range of pharmacological activities and therapeutic benefits including anti‐inflammatory, anticancer, antimicrobial, antioxidant, and wound healing effect (Williams et al. 2011). In particular, curcumin has been widely investigated in cellular and animal models of neuroinflammation. Experiments using BV-2 microglia revealed that this diarylheptanoid inhibited neuroinflammation in LTA-activated microglial cells through reduction in the production of TNFα, PGE_2_, and nitric oxide (NO), as well as inhibition of NF-κB and MAPK activation (Yu et al. 2018). Similar observations were made in BV-2 microglia stimulated with LPS (Porro et al. 2019; Zhang et al. 2019). These observations in the microglia have been confirmed by results of experiments in animal models of AD which showed that curcumin treatment ameliorated cognitive decline and neuroinflammation following exposure to LPS, and in p25 transgenic mice (Sundaram et al. 2017; Sorrenti et al. 2018). Curcumin also inhibited formation of amyloid beta oligomers and fibrils and reduced amyloid in mouse models of AD (Yang et al. 2005).

Interestingly, a clinical trial with curcumin did not demonstrate efficacy in AD in a 24-week placebo-controlled trial (Ringman et al. 2012). The authors suggested that the lack of efficacy could be related to bioavailability of the product used or differences in the biology of rodent models of AD and human AD. However, a subsequent randomised, double-blind, placebo-controlled study in healthy older population which employed 400 mg/day of a highly bioavailable curcumin preparation (Longvida), reported a significantly improved working memory and mood after a 4-week treatment (Cox et al. 2015). These studies suggest that the clinical efficacy of curcumin in AD would be increased by approaches which enhance its bioavailability.

Our investigations revealed that other polyphenols such as formononetin, an isoflavone in *Trifolium pratense* (red clover) inhibited neuroinflammation through mechanisms involving attenuation of NF-κB activation in LPS-activated BV-2 microglia (El-Bakoush and Olajide 2018). Similar results were obtained in experiments on agathisflavone, a biflavonoid isolated from *Anacardium occidentale* (Velagapudi et al. 2018a, b). These observations have not been confirmed in animal experiments. It is expected that future animal experiments to establish in vivo activities of these compounds would be valuable in determining their potentials for follow-up clinical studies.

Flavonoids remain one of the important groups of natural products for inhibiting neuroinflammation in AD due to their fundamental inhibitory actions on pro-inflammatory transcription factors. Furthermore, this group of compounds activate antioxidant/anti-inflammatory transcription factors. While flavonoids have proven to be promising therapeutic natural products in pre-clinical models of AD, it is important to note that the overall bioavailability of parent flavonoids are usually low. Furthermore, flavonoids do not cross the blood–brain barrier easily due to their high polarity.

### Terpenes

**Fig. 3:**
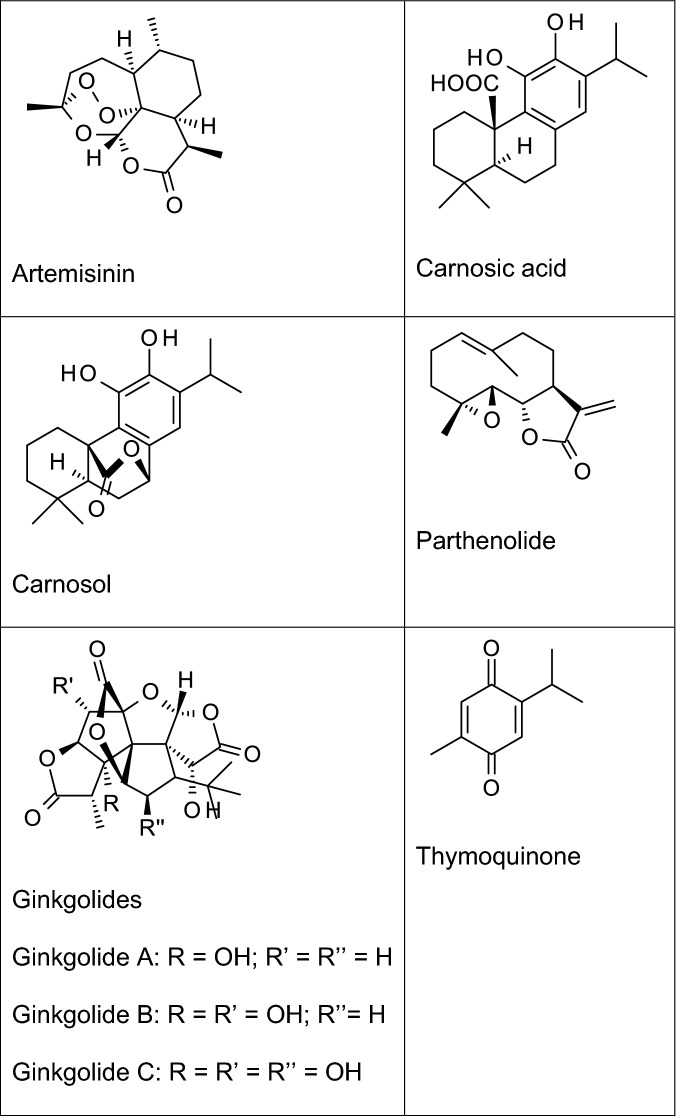
Examples of some anti-neuroinflammatory terpenoids

Terpenoids are a large and structurally diverse group of compounds formed biosynthetically from a combination of two or more isoprene units (a five carbon unit, chemically known as 2-methyl-1,3-butadiene) (Nahar and Sarker 2019). Terpenoids (Fig. 3) have been widely reported to inhibit neuroinflammation in animal models and in vitro.

Parthenolide, a biologically-active sesquiterpene lactone present in *Tanacetum parthenium* has been reported to improve cognitive function and decrease levels of TNF-α and IL-6 in the cortical and hippocampal regions of rats (Khare et al. 2017). Recently, this compound was reported to inhibit neuroinflammation in intracerebral haemorrhage-induced brain injury in rats through TLR4/NF-κB-mediated reduction in the levels of TNF-α, interleukin IL-6, IL-17 in the ipsilateral hemispheres of the brain (Wang et al. 2020). It appears that the NF-κB inhibitory action of parthenolide is responsible for its versatile inhibitory actions in different neuropathologies involving inflammation. Neuroprotection by this sesquiterpene lactone needs to be further confirmed in AD clinical trials.

Artemisinin is a sesquiterpene lactone found in the Chinese herb *Artemisia annua* (Qinghao) of the family Asteraceae, and was originally developed for the treatment of multi-drug resistant malaria. Recently, this compound and some of its synthetic analogues have been reported to possess potential neuroprotective activity in AD, partly through their anti-inflammatory activity. Studies reported by Zhu et al. (2012) revealed that artemisinin inhibited LPS-induced release of TNFα, IL-6, MCP-1 and NO in BV-2 microglia. These authors further suggest that the observed inhibition of neuroinflammation by artemisinin was related to its modulatory effects on the NF-κB signalling pathway in the microglia. Subsequent studies showed that this compound was neuroprotective in a mouse model of AD through reduction in the levels of IL-1β, IL-6 and TNF-α in the hippocampus and the cortex (Qiang et al. 2018). Similarly, we have reported that artemisinin analogues, artesunate and artemether inhibited neuroinflammation by targeting NF-κB signalling in BV-2 microglia (Okorji and Olajide, 2014; Okorji et al. 2016). Artemisinin and its synthetic derivatives have been shown to cross the blood–brain barrier due to their lipophilicity (Navaratnam et al. 2000). However, experiments in animals have suggested that the compounds are neurotoxic (Meshnick 2002; Genovese and Newman, 2008), which may be discouraging their investigation in AD clinical trials.

Our investigations have shown that thymoquinone (the main bioactive constituent of *Nigella sativa*) is a potent inhibitor of neuroinflammation. In LPS-activated BV-2 microglia, thymoquinone treatment resulted in significant reduction in TNFα, IL-6, PGE_2_, and NO protein and mRNA through mechanisms involving inhibition of the pro-inflammatory NF-κB and activation of the anti-inflammatory Nrf2 pathways (Velagapudi et al. 2017b). We further showed that inhibition of neuroinflammation by this compound was partially related to activation of both sirtuin 1 (SIRT-1) and 5′ adenosine monophosphate-activated protein kinase (AMPK) in the microglia (Velagapudi et al. 2017a). Similar observations showing inhibition of neuroinflammation by thymoquinone were made in recent investigations reported by Cobourne-Duval et al. (2018). Recently, thymoquinone was shown to improve cognitive decline in a rat model of AD, while decreasing Aβ formation and accumulation, as well as TNF-α and IL-1β (Abulfadl et al. 2018).

Carnosic acid and carnosol are brain-permeable natural diterpenes found in *Rosmarinus officinalis*, and have shown significant neuroprotective activity (de Oliveira, 2016). Studies by Foresti et al. (2013) showed that carnosol could inhibit neuroinflammation in BV-2 microglia by reducing levels of TNF-α, PGE_2_ and nitric oxide following activation with either LPS or interferon gamma (IFNγ). In addition, carnosic acid was reported to produce anti-inflammatory effect in paraquat-induced increase in the levels of IL-1β, TNFα, and cyclooxygenase-2 (COX-2) in SH-SY5Y cells by targeting Nrf2/HO-1 and NF-κB signalling pathways (de Oliveira et al. 2018). It appears that inhibition of neuroinflammation by carnosol and carnosic acid could be related to Nrf2 activation, which is known to result in an anti-inflammatory outcome (Innamorato et al. 2008). Ginkgolides are pharmacologically-active diterpenes found in *Ginkgo biloba* (Ginkgoaceae). In a study reported by Zhou et al. (2016), ginkgolides were shown to inhibit neuroinflammation by reducing levels of IL-1β, IL-6, IL-8, TNF-α in BV-2 microglia activated with oxygen–glucose deprivation and re-oxygenation through mechanisms involving TLRs/MyD88/NF-κB signalling pathways. Anti-inflammatory activity has also been reported to contribute to the neuroprotective actions of ginkgolides in models of cerebral ischemia and reperfusion injury (Gu et al. 2012; Jiang et al. 2014).

There are no reports in literature indicating the benefits of ginkgolides in clinical trials for AD treatment. Clinical studies on standardised *Ginkgo biloba* extracts have shown conflicting results. Results of a randomised controlled trial to evaluate the efficacy of Ginkgo biloba extract (240 mg) in patients diagnosed with mild to AD or vascular dementia showed that the extract improved cognitive function, neuropsychiatric symptoms and functional abilities (Ihl et al. 2012). However, in another study to assess the efficacy of long-term use of Ginkgo biloba extract (120 mg) for the reduction of incidence of AD in elderly adults with memory complaints, the extract did not reduce the risk of progression to AD when compared with placebo (Vellas et al. 2012). A systematic review and meta-analysis of randomised controlled trials of *Ginkgo biloba* extract in mild cognitive impairment and AD attributed the conflicting outcomes of the trials to limited sample size, inconsistent findings and methodological quality of included trials (Yang et al. 2016). Furthermore*,* the effectiveness of Ginkgo biloba extract in treating established AD without preventing its incidence warrants further investigation.

### Carotenoids from Crocus sativus (Saffron)

*Crocus sativus* (Saffron) is a spice that is widely reputed for a wide variety of therapeutic applications, including neurodegenerative disorders, depression, diabetes mellitus, atherosclerosis and cancer (Leone et al. 2018). Evidence from in vitro experiments, animal models of AD and clinical trials have shown that carotenoids in saffron flowers, crocin and crocetin are neuroprotective natural products with therapeutic potentials in AD. Studies in BV-2 microglia showed that crocin and crocetin inhibited LPS-induced production of NO/iNOS, TNF-α, IL-1β and ROS in BV-2 microglial cells through mechanisms linked to NF-κB (Nam et al. 2010; Zhang et al. 2018). These anti-inflammatory carotenoids have been reported to produce promising activities in animal models of AD through their ability to improve cognitive function (Hosseinzadeh et al. 2012; Asadi et al. 2015). A study published by Mazumder et al. (2017) appears to provide a link between the anti-inflammatory and antioxidant activities of crocin and its ability to enhance cognitive abilities in mice. This hypothesis needs to be further investigated to provide a better understanding of how anti-inflammatory natural products promote cognitive abilities.

There are no clinical studies to demonstrate the benefits of crocin and crocetin in AD. However, results of single- and double-blind, placebo controlled clinical trials on saffron have shown promising effects in patients with moderate to severe Alzheimer's disease (Akhondzadeh et al. 2010a, b; Farokhnia et al. 2014; Tsolaki et al. 2016).

### Marine natural products

The most investigated neuroprotective marine natural product is astaxanthin (3,3′-dihydroxy-β,β′-carotene-4,4′-dione), a xanthophyll carotenoid found in *Haematococcus pluvialis*, *Chlorella zofingiensis*, *Chlorococcum*, and *Phaffia rhodozyma*. In LPS-stimulated BV-2 microglia, astaxanthin was reported to inhibit NO/iNOS and COX-2 (Choi et al. 2008). In a separate study, Kim et al. (2010a, b) showed that the compound attenuated LPS-induced production of IL-6 in BV-2 microglia through mechanisms involving ERK1/2-MSK-1 and NF-κB activation.

Inhibition of neuroinflammation has also been reported for astaxanthin in animal models of neurodegeneration. In a study reported by Zhou et al. (2015), treatment of diabetic mice with astaxanthin alleviated alleviated cognition deficits with accompanying inhibition in NF-κB mediated neuroinflammation. Inhibition of neuroinflammation was also proposed to be responsible for the neuroprotection by astaxanthin in in experimental subarachnoid haemorrhage (Zhang et al. 2014). Interestingly, clinical trials on the benefits of astaxanthin in improving cognition have shown promising results (Satoh et al. 2009; Katagiri et al. 2012), suggesting that this marine natural product holds significant promise in the development of novel therapeutics for neurodegenerative disorders such as AD.

### Challenges in the delivery of natural products to the brain: novel delivery technologies

Treatment of AD is challenging partly due to the presence physical barriers such as the blood–brain barrier (BBB) in the brain. The BBB is the critical barrier that needs to be overcome to transport natural compounds from the blood into brain. The challenges posed by the BBB is important in the activity of neuroprotective natural products because their benefit is significantly affected by low bioavailability and sometimes poor pharmacokinetic profile (Manach et al. 2004; Soares et al. 2018).

The bioavailability of some anti-inflammatory natural products is attributed to their metabolism. Curcumin is a widely-studied anti-inflammatory and antioxidant polyphenol. However, the pharmacological potential is restricted because of its low bioavailability following oral administration (Aggarwal and Sung 2009; Kumar et al. 2010; Di Meo et al. 2019). Resveratrol is a polyphenol with antioxidant, anti-inflammatory and neuroprotective actions. However, in spite of its lipophilicity resveratrol is extensively metabolised and rapidly eliminated resulting in poor bioavailability (Chimento et al. 2019). The poor bioavailability profiles of these polyphenols have triggered the synthesis of more bioavailable derivatives.

Delivery technologies involving mostly lipid-based nanocarriers have been explored to enhance the bioavailability and BBB penetration of neuroprotective natural products. For example, liposomes are formed by amphiphilic substances such as phospholipids that self-assemble as vesicles which compose of lipid bilayers (Lúcio et al. 2010). Over the years, lipid nanoparticles have become more popular and have the advantage of protecting active compounds (Soares et al. 2018). Solid lipid nanoparticles (SLNs) are a new generation of submicron-sized lipid emulsions in which the liquid lipid (oil) has been substituted by a solid lipid with an ability to penetrate the BBB and produce a pharmacological action in the CNS (Mutoh et al. 2016). Table 1 summarises the novel delivery strategies which have been applied in enhancing the permeability of some anti-inflammatory natural products into the brain.

**Table 1:** Nanocarriers for delivering natural products into the brain

Natural product	Delivery strategy	References
Curcumin	Micelles	Hagl et al. (2015)
	Liposomes	Mourtas et al. (2014)
	Nanoemulsion	Sood et al. (2014)
	Solid lipid nanoparticles	Kakkar and Kaur (2011)
	Nanostructured lipid carriers	Puglia et al. (2012)
EGCG	Nanoemulsion	Barras et al. (2009)
	Lipid nanoparticles	Smith et al. (2010)
Luteolin	Solid lipid nanoparticles	Dang et al. (2014)
	Liposomes	Zhao et al. (2011)
Thymoquinone	Nanoemulsion	Ahmad et al. (2016)
	Solid lipid nanoparticles	Ramachandran and Thangarajan (2016)
Resveratrol	Liposomes	Wang et al. (2011)

## Conclusion and future direction

Natural products exhibit promising health-promoting effects in neurodegenerative diseases partly due to their anti-inflammatory property. The emerging research data on the possible therapeutic effects of natural products as neuroprotective agents is particularly exciting due to the steadily increasing population with AD.

In spite of the overwhelming evidence suggesting the potentials of anti-inflammatory natural products in treating AD, further investigations are required to assess their clinical efficacy in properly-controlled human trials. Studies have shown that in vitro data demonstrating efficacy do not always translate into in vivo effects. Furthermore, extrapolating data from animal models to humans in the search for new treatment for AD is not reliable due to significant species differences. To overcome this challenge, pre-clinical investigations on anti-inflammatory natural products need to focus on employing new cutting-edge tools. One of such approaches is the use of a tri-culture including human neurons, astrocytes and microglia to evaluate neuroinflammation and neuroprotection in vitro.

Small molecules for AD must be bioavailable and overcome the challenges posed by the BBB to act in the brain. Published articles have shown the potential values of nanocarriers in delivering natural products to the brain. More research efforts need to focus on new delivery methods to achieve significant therapeutic concentrations of polar phytochemicals (such as the anti-inflammatory/antioxidant polyphenols) in the brain.

**Fig. 4:**
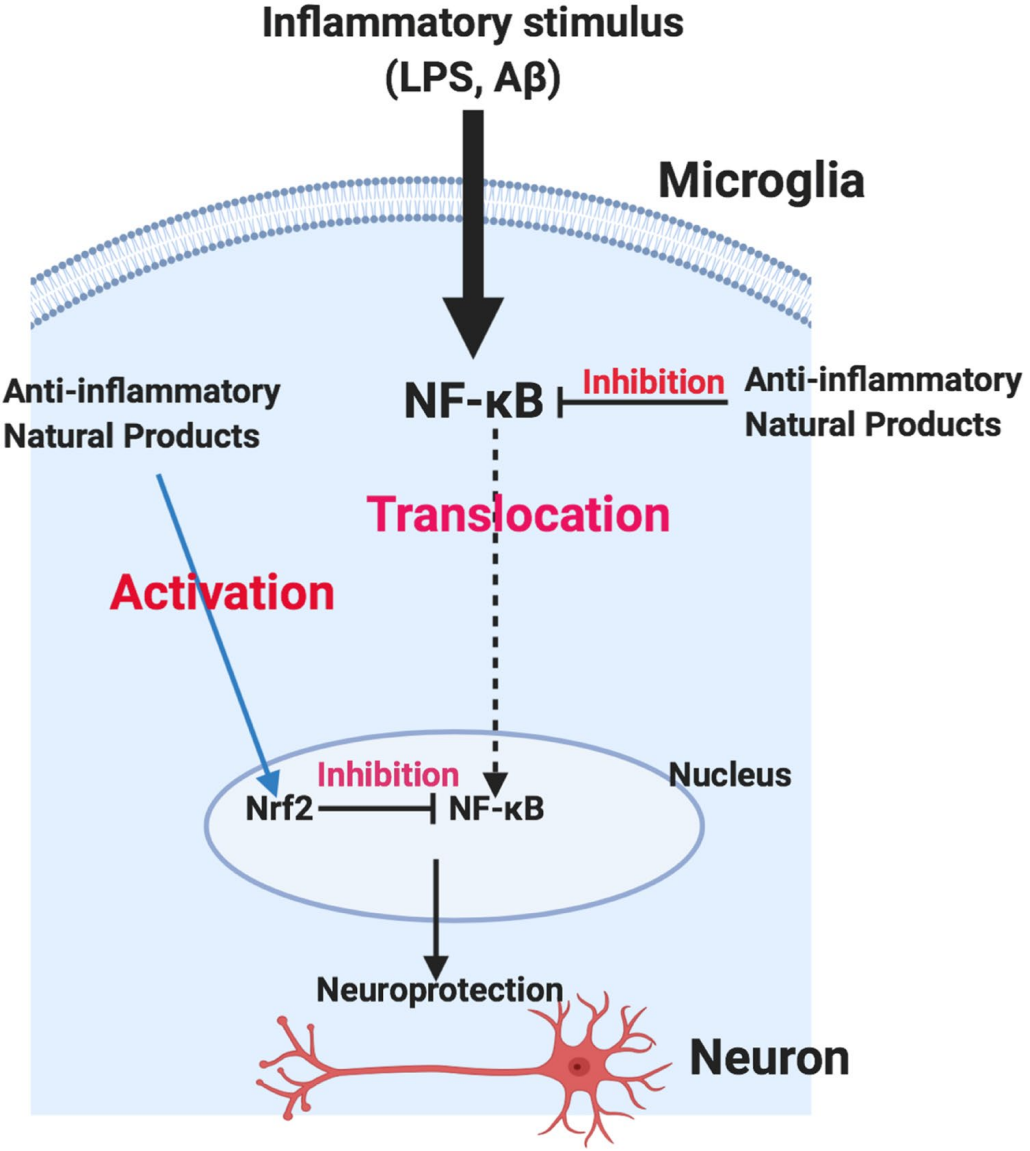
Summary of molecular targets of anti-neuroinflammatory natural products action involving the transcription factors NF-κB and Nrf2

With regards to molecular target-driven discovery of novel natural products for AD, focusing on a single gene is not the best disease model; as most pharmacologically-active natural products identified using this approach have not resulted in new treatments, mainly due to the complex mechanisms involved in AD. Investigations need to focus on the two principal transcription factors, NF-κB and Nrf2, which control key molecular players in producing inflammation or anti-inflammation (Fig. 4). Furthermore, pre-clinical investigations on AD should focus more on experimental models which combine stress reduction (neuroinflammation, oxidative stress), neuroprotection, and regeneration.
